# IgG4-Related Disease Presenting as Recurrent Mastoiditis With Central Nervous System Involvement

**DOI:** 10.1177/2324709614553670

**Published:** 2014-09-26

**Authors:** April L. Barnado, Melissa A. Cunningham

**Affiliations:** 1 Medical University of South Carolina, Charleston, SC, USA

**Keywords:** IgG4-related disease, mastoiditis, inflammatory pseudotumor

## Abstract

We report a case of a 43-year-old female who presented with right ear fullness and otorrhea. She was initially diagnosed with mastoiditis that was not responsive to multiple courses of antibiotics and steroids. She was then diagnosed with refractory inflammatory pseudotumor, and subsequent treatments included several mastoidectomies, further steroids, and radiation therapy. The patient went on to develop mastoiditis on the contralateral side as well as central nervous system involvement with headaches and right-sided facial paresthesias. Reexamination of the mastoid tissue revealed a significantly increased number of IgG4-positive cells, suggesting a diagnosis of IgG4-related disease. The patient improved clinically and radiographically with rituximab and was able to taper off azathioprine and prednisone. IgG4-related disease should be considered in patients with otologic symptoms and be on the differential diagnosis in patients with inflammatory pseudotumor. Staining for IgG and IgG4 is essential to ensure a prompt diagnosis and treatment.

## Introduction

IgG4-related disease is a recently recognized entity initially described in patients with autoimmune pancreatitis. It is now characterized as a systemic condition that can affect every organ system, often with elevated serum IgG4 concentrations, with characteristic pathologic features including predominance of IgG4-positive plasma cells in tissue, storiform fibrosis, obliterative phlebitis, and an eosinophilic infiltrate.^1^ IgG4-related disease has been most commonly described affecting the salivary glands, submandibular glands, and pancreas. However, there have been rare case reports describing central nervous system (CNS) involvement involving the meninges as well as inflammatory pseudotumor formation. In addition, otologic manifestations have been described but are very rare, with only one case report describing involvement of the mastoid.^2^ Herein, we describe a patient with IgG4-related disease who presented with recurrent mastoiditis as well as CNS involvement resulting in multiple mastoidectomies and radiation therapy prior to her diagnosis and successful treatment with rituximab.

## Case Presentation

A 43-year-old female with no significant past medical history presented with right ear fullness, pressure, and otorrhea. She was diagnosed with mastoiditis and treated with antibiotics and steroids but subsequently developed right-sided tinnitus and conductive hearing loss. Three months later, she had a right eustachian tube placed for otorrhea with initial improvement in her symptoms. However, her symptoms recurred with computed tomography (CT) of the temporal bone showing right-sided coalescent mastoiditis. The initial differential diagnosis included bacterial infection, lymphoma, and ANCA-associated vasculitis. Laboratory evaluation revealed no leukocytosis with normal differential, elevated sedimentation rate (ESR) at 41, and a normal C-reactive protein (CRP). An ANCA was negative. The patient then underwent a simple mastoidectomy with intraoperative findings of boney destruction of mastoid cortex and air cells. Pathologic studies revealed negative bacterial culture with a dense lymphoplasmacytic infiltrate and fibrosis that was reported as chronic mastoiditis. There was no evidence for malignancy with no cellular atypia or pleomorphism, immunohistochemistry staining showing polyclonal plasmacytosis, and negative flow cytometry. As infection and malignancy were excluded, it was felt that the patient’s lesion was most likely inflammatory pseudotumor.

**Figure 1.**
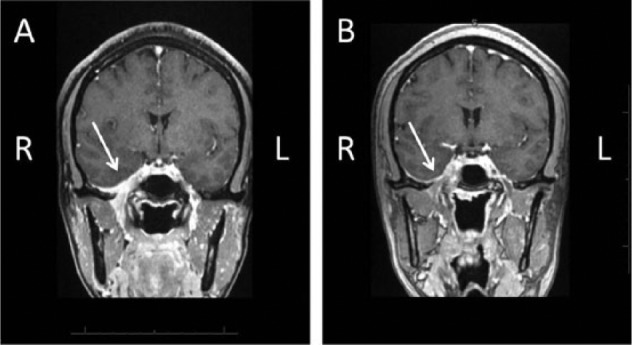
MRI internal auditory canals with and without contrast, axial view, showing confluent areas of enhancement of the right mastoid (arrow) and involvement of left mastoid (double arrow).

The patient’s symptoms initially improved after her resection but again recurred within several months. Magnetic resonance imaging (MRI) of the auditory canal at that time showed increased enhancement in the mastoid bowl and the eustachian tube consistent with right middle ear involvement (Figure 1). The patient received prednisone up to 40 mg daily for suspected inflammatory pseudotumor with partial but not complete resolution of symptoms. Due to incomplete symptom response to multiple steroid courses, the patient had a repeat resection of the pseudotumor from the right skull base with mastoidectomy and ossicular reconstruction. Pathology from this resection revealed findings similar to the prior resection (chronic mastoiditis with no evidence of malignancy). With persistent symptoms despite corticosteroids and 2 resections, the patient then underwent a course of radiation to the right temporal region to treat presumed refractory inflammatory pseudotumor.

While the patient’s right-sided tinnitus, otorrhea, and hearing loss stabilized, over the next 9 months, she developed similar symptoms affecting the left side including tinnitus and hearing loss. MRI of the internal auditory canal revealed no recurrence of the patient’s right-sided disease but showed opacification of the left mastoid air cells. It was felt that the patient was now developing left-sided mastoiditis with suspicion for inflammatory pseudotumor. The patient underwent resection of the left mastoid and skull base. Similar to the pathology from the prior 2 resections, the tissue was again benign, with a dense lymphoplasmacytic infiltrate and fibrosis that was reported as chronic mastoiditis.

**Figure 2.**
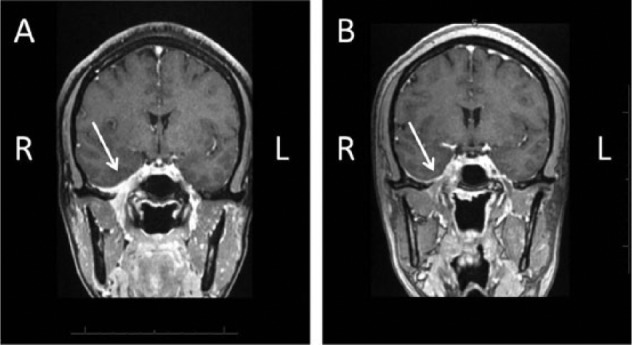
(A) MRI brain and orbits with and without contrast, coronal view, showing right-sided dural enhancement (arrow) of the middle cranial fossa, cavernous sinus, and foramen ovale. (B) MRI brain and orbits with and without contrast, coronal view, showing improvement in right-sided dural enhancement (arrow) of the middle cranial fossa after steroids.

Despite resection, the patient’s symptoms evolved to severe, daily headaches that occurred at the medial end of the right orbit and were associated with loss of taste and right-sided facial paresthesias. Workup for the new-onset, severe headaches included a lumbar puncture with a normal white blood cell count, protein, and glucose with negative bacterial and fungal cultures. Cerebrospinal fluid was significant for positive oligoclonal banding, as well as a mildly elevated IgG index (0.88) and IgG synthesis rate (7.9). MRI of the brain showed progression of prior right-sided middle ear and skull base disease with enhancement of the geniculate ganglion of the right facial nerve along with involvement of the right temporal horn (Figure 2A). The patient was given pulse doses of methylprednisolone with improvement in headache, right-sided facial paresthesias, and loss of taste. In addition, her brain MRI revealed improvement of her CNS and middle cranial fossa disease (Figure 2B). However, as her steroids were tapered, symptoms recurred. The patient was started on azathioprine as a steroid-sparing agent with incomplete relief of symptoms with tapering of steroids.

At this time, it was felt that the patient’s disease was refractory inflammatory pseudotumor versus possible neurosarcoidosis. However, the patient had no other signs or symptoms consistent with sarcoidosis. Chest imaging was normal, and review of pathology from multiple resections revealed no granulomas. The patient, whose family history included thyroid disease and systemic lupus erythematosus in her mother, was referred for evaluation in the rheumatology clinic regarding possible underlying autoimmune disease versus neurosarcoidosis. Her review of systems was significant for mild dry mouth, headaches, and neurologic symptoms (mild right-sided facial paresthesias) that had overall improved. Her exam was normal including a nonfocal neurologic exam. Given the clinical history and the diagnosis of inflammatory pseudotumor, there was a suspicion for IgG4-related disease. Pathology was reevaluated with IgG4 staining of the right mastoid tissue, which showed an increased number of IgG4-positive cells, up to 200 per high power field. In addition, further review of the pathology revealed characteristic histologic features of IgG4-related disease with dense lymphoplasmacytic infiltrate, storiform fibrosis, and obliterative phlebitis. A second opinion was obtained at The IgG4-related Systemic Disease Program at The Massachusetts General Hospital, where Dr John Stone and his team evaluated the patient, reviewed the pathology, and concurred with the diagnosis and treatment plan. The patient was started on rituximab 1000 mg intravenous every 2 weeks for 2 doses with plan for redosing in 6 months. With starting rituximab, the patient was able to discontinue prednisone and azathioprine with stabilization of symptoms and no recurrence found on imaging.

## Discussion

As knowledge has increased about IgG4-related disease, there are increasing reports of new organ involvement not previously described. Diagnostic terminology has been proposed with cases being considered highly suggestive for IgG4-related disease if at least 2 of the characteristic histologic features of (*a*) dense lymphoplasmacytic infiltrate, (*b*) storiform fibrosis, and (*c*) obliterative phlebitis are present as well as increased IgG4 tissue counts.^3^ Our case’s mastoid tissue had all 3 histologic features as well as an elevated number of IgG4 plasma cells establishing the diagnosis.

Prior literature and our case highlight the increasing identification of IgG4-related disease presenting with disease affecting the sinuses and the ear. Our case is unique in that it represents the second case to date of IgG4-related disease presenting as recurrent mastoiditis. In addition, our case is unique with its progression from middle ear disease to CNS disease. There is one other case report of a patient who initially presented with an ear effusion and otorrhea and then had a CT revealing mastoiditis.^2^ This patient underwent a mastoidectomy with similar pathologic findings to our case with negative stains for infectious agents and benign inflammatory tissue. The patient also developed CNS disease with brain MRI showing left cerebritis and later developed mastoiditis on the contralateral side requiring a second mastoidectomy. In contrast to our case, however, this patient improved clinically and radiographically with prednisone monotherapy.

In reviewing the literature of IgG4-related disease with mastoiditis, in addition to the above case, there was a 2009 case report.^4^ However, this case did not clearly establish if the mastoiditis was due to the patient’s underlying IgG4-related disease or prior history of chronic infections.^4^ In reviewing otologic manifestations of IgG4-related disease, there were 3 additional case reports.^5-7^ One case report included a patient with IgG4-related disease with inner ear involvement presenting with mixed hearing loss and a middle ear effusion in addition to renal, lung, and lacrimal gland involvement.^5^ A second case report described a patient presenting with autoimmune pancreatitis accompanied by sclerosing sialadenitis, allergic purpura, endocapillary proliferative glomerulonephritis, and autoimmune sensorineural hearing loss. This patient had positive immunostaining for IgG4 in the salivary gland and kidney, so it was assumed that the hearing loss, as well as other systemic involvement, was related to IgG4-related disease.^6^ Last, the third case was a 58-year-old woman with multiple inflammatory pseudotumors involving the pharynx, gall bladder, lungs, pelvis, omentum, eyes, and left temporal bone. The patient had intermittent hearing loss and was found to have a completely opacified left middle ear cleft and mastoid.^7^

Our patient was initially diagnosed as having inflammatory pseudotumor, which has been increasingly identified as IgG4-related disease, particularly disease involving the CNS. The diagnosis of inflammatory pseudotumor has been applied to a heterogeneous group of disease entities with mass-forming lesions with an inflammatory infiltrate composed predominantly of lymphocytes and plasma cells. Inflammatory pseudotumor was first described in the lung and most commonly involves the lung and liver.^8^ CNS lesions are rare and usually arise from the meninges with many lesions reported as dural arising from the skull base.^8,9^ One case series reports 16 potential cases of meningeal IgG4-related disease.^10^ Similar to these patients, our patient had skull base involvement and developed severe headache with focal neurologic deficits related to facial nerve involvement. Of note, many of the IgG4-related disease cases involving the CNS are limited to the CNS without evidence of other organ involvement, as was true in our patient.^9^

Similar to other reports of IgG4-related disease, our patient’s symptoms and imaging did improve with steroids. However, our patient had recurrence of symptoms and development of new lesions on tapering steroids, despite the addition of immunosuppressive therapy with azathioprine. While many patients are initially responsive to steroids, relapses can occur requiring additional immunosuppression such as azathioprine, methotrexate, or mycophenolate mofetil. Additionally, rituximab has been used in relapsing and refractory cases with the benefit of a quick clinical response and a high rate of efficacy.^11^

In summary, we describe a second case of IgG4-related disease presenting as recurrent mastoiditis that progressed to CNS involvement with significant symptomology. Our case illustrates that IgG4-related disease can involve the ear and should be in the differential when considering autoimmune causes of hearing loss, such as ANCA-associated vasculitis. IgG4-related disease should also be on the differential with CNS lesions described as inflammatory pseudotumor. With the suspicion for IgG4-related disease, staining for IgG and IgG4 is essential to ensure a prompt diagnosis and treatment.
